# Efficient Perovskite/Silicon Tandem Solar Cells Using Hybrid Two‐Step Inkjet Printing with Edge Isolation Precision

**DOI:** 10.1002/smsc.202500362

**Published:** 2025-09-23

**Authors:** Raphael Pesch, Julian Petry, Julian Petermann, Ronja Pappenberger, Theresa Kuechle, Johannes Schenck, Lena Paula Rothbauer, Lingyi Fang, Xuzheng Liu, Saeid Rafizadeh, Bahram Abdollahi Nejand, Johannes Sutter, Ulrich Lemmer, Ulrich Wilhelm Paetzold

**Affiliations:** ^1^ Institute of Microstructure Technology (IMT) Karlsruhe Institute of Technology (KIT) Hermann‐von‐ Helmholtz‐Platz 1 76344 Eggenstein‐Leopoldshafen Germany; ^2^ Light Technology Institute (LTI) Karlsruhe Institute of Technology (KIT) Engesserstrasse 13 76131 Karlsruhe Germany; ^3^ Department of Research and Development Meyer Burger Technology A G An d. Baumschule 6‐8 09337 Hohenstein‐Ernstthal Germany

**Keywords:** hybrid deposition, inkjet printing, perovskite, tandem solar cell, two‐step deposition

## Abstract

Developing high‐efficiency perovskite/silicon tandem solar cells (PSTs) using scalable deposition methods is crucial for the industrialization of next‐generation photovoltaics. However, developing industrially viable deposition techniques to ensure high performance, uniformity, and compatibility with existing silicon manufacturing remains a key challenge. A scalable hybrid two‐step deposition process, combining evaporation and inkjet printing, is presented for fabrication of high‐performance PSTs. Wide bandgap perovskite solar cells are achieved with power conversion efficiencies (PCEs) of up to 19.8%. Applying this approach to textured silicon bottom cells, the process ensures conformal perovskite growth, critical for industry‐relevant tandem integration. Using this technique, highly efficient, fully textured PSTs with a PCE of 27.4% are fabricated. Homogeneous perovskite thin films are formed up to the substrate's very edge, enabling industry standards for silicon edge isolation. These results highlight the potential of hybrid two‐step inkjet printing for scalable, high‐efficiency PST fabrication, paving the way for industrial adoption.

## Graphical abstract

Crucial advancements are still needed to enable scalable, reliable fabrication of perovskite photovoltaics. A hybrid evaporation and inkjet printing method is introduced to produce high‐efficiency perovskite/silicon tandem solar cells. The process achieves 27.4% power conversion efficiency on fully textured silicon with uniform, edge‐to‐edge coverage, meeting industry standards and offering strong potential for large‐scale commercial solar cell production.

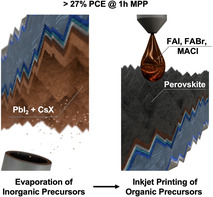

## 1 Introduction

Although perovskite top solar cells are promising candidates for silicon‐based tandem photovoltaics, scaling up the deposition process while maintaining high‐quality thin films remains challenging.^[^ ^1^ ^]^ Over the past decade, several scaling techniques, such as slot‐die coating, evaporation, spray coating, and inkjet printing, have been identified as suitable candidates for upscaling.^[^ ^2^, ^3^, ^4^, ^5^ ^]^ Among these various available technologies, inkjet printing stands out as the only method that allows for free‐form fabrication, provides the most efficient material usage, and enables precise spatial deposition control.^[^ ^6^, ^7^ ^]^ Additionally, this technique has already been applied in industrial manufacturing, such as the production of organic light‐emitting diodes.^[^ ^8^ ^]^ While early‐stage perovskite development focused on one‐step deposition, two‐step deposition, which separates the deposition of the inorganic and organic compounds into two consecutive steps, has become equally important for perovskite solar cell fabrication.^[^ ^9^, ^10^ ^]^ Its major advantages include high operability and good performance reproducibility.^[^ ^11^ ^]^ In perovskite/silicon tandem solar cells (PSTs), the perovskite thin film is deposited on top of the silicon solar cell with a wide bandgap of around 1.70 eV, enabling the highest efficiencies in optical simulations.^[^ ^12^ ^]^ Since all industrially relevant silicon solar cells exhibit micrometer‐sized, pyramid‐shaped texture through etching, depositing uniform and high‐quality perovskite thin films to this texture is crucial.^[^ ^13^, ^14^ ^]^ This can be accomplished by either increasing the thickness of the perovskite to adequately cover the pyramids or by choosing a deposition technique that guarantees a conformal coating.^[^ ^15^, ^16^ ^]^ Using a thick, nonconformal perovskite to cover the entire texture results in a laterally varying perovskite thickness, which is also susceptible to shunts if some pyramids are not fully covered.^[^ ^17^ ^]^ Furthermore, the etching process occurs randomly and is hence hard to control. Fabricating sub‐micrometer textured silicon, which could be covered by nonconformal deposited perovskite, is more complex and additionally shows higher reflection losses compared to fully textured devices.^[^ ^18^ ^]^ In contrast, a deposition technique producing conformal perovskite thin films maintains the pyramid‐shaped, reflection‐suppressing texture.^[^ ^13^, ^16^ ^]^ Evaporation‐based deposition techniques have already been shown to enable conformal growth.^[^ ^19^ ^]^ Since fully evaporation‐based perovskite deposition remains challenging, this technique requires further optimization to achieve results compatible with current solution‐processed champion PSTs. A promising method that also preserves conformability is the hybrid two‐step deposition, where inorganic precursors are evaporated, and organic precursors are solution‐processed.^[^ ^16^ ^]^ This technique has produced certified PSTs with power conversion efficiencies (PCEs) exceeding 31%.^[^ ^13^, ^16^ ^]^ However, as the second step, the nonscalable spin‐coating technique was employed to achieve this.

To this end, we demonstrate inkjet printing as a scalable alternative to the nonscalable spin‐coating step in the hybrid two‐step perovskite deposition process. We show that hybrid two‐step deposition is a promising process for minimizing material waste, with a theoretical material yield of 100% for the organic precursor materials. By fine‐tuning the inkjet printing parameters and using surface passivation, we demonstrate a highly efficient wide bandgap single‐junction PSC and a fully textured PST with a PCE of 19.8% and 27.4%, respectively. To the best of our knowledge, this is the highest reported PCE for inkjet‐printed, hybrid two‐step processed PSTs. Lastly, we demonstrate that our hybrid process solves a widely overlooked problem when scaling the process to full wafer size. We introduce inkjet printing as a solution‐based deposition method capable of depositing a high‐quality perovskite thin film up to the solar cell's edge. For the photovoltaic industry, this is crucial, as edge isolation is necessary for silicon solar cells and, by extension, PSTs.^[^ ^20^, ^21^ ^]^ Furthermore, this allows for maximizing the active area of PSTs, bringing it as close as possible to that of the silicon bottom cells.

## 2 Results and Discussion

**Figure 1:**
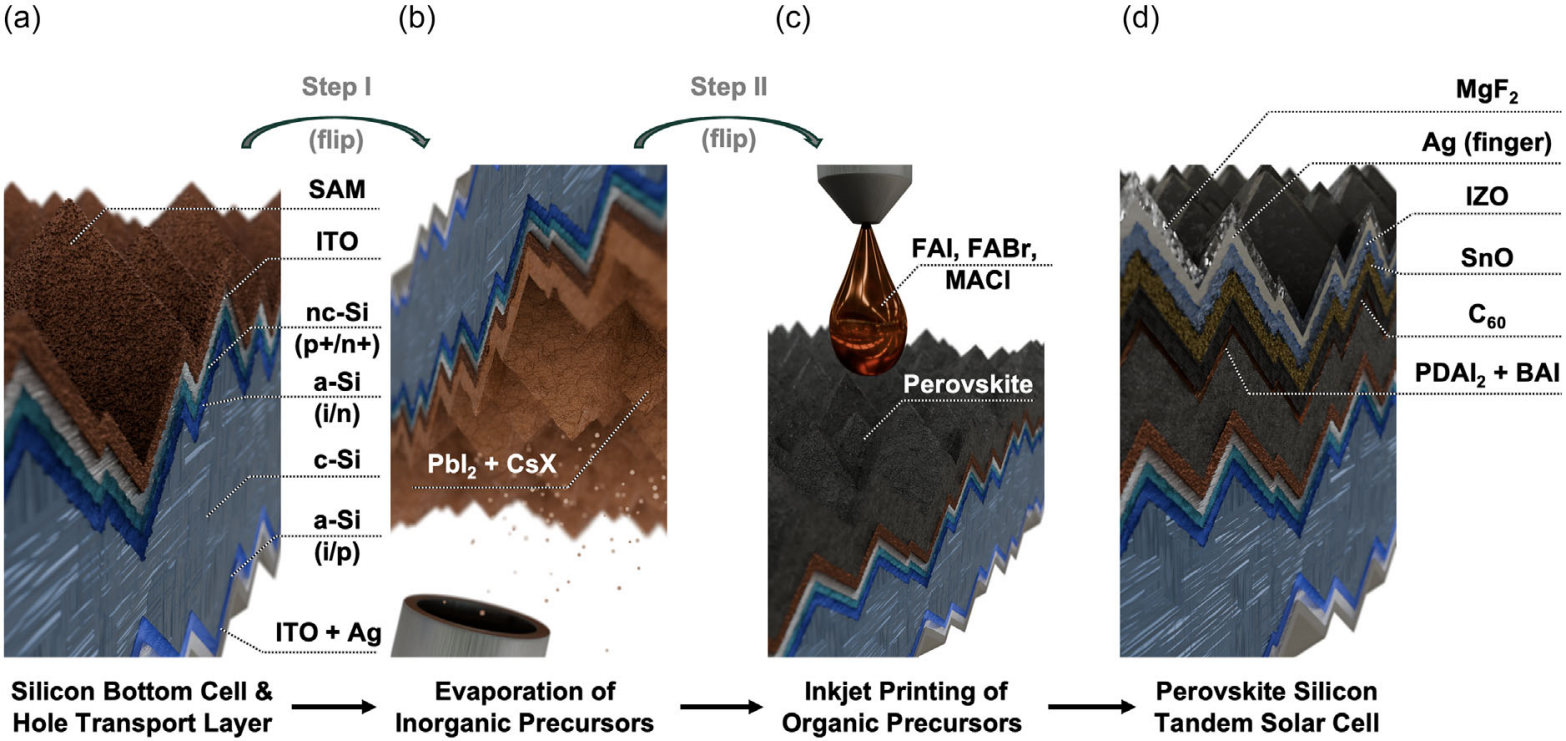
Fabrication process of PSTs using a hybrid two‐step deposition method that combines evaporation and inkjet printing. a) Starting from silicon bottom solar cells coated with the HTL, b) the inorganic thin film is applied by evaporating lead iodide together with cesium bromide or cesium chloride. c) For perovskite formation, the organic precursors formamidinium iodide, formamidinium bromide, and methylammonium chloride are then deposited using inkjet printing. d) Finally, surface passivation, the ETL, a buffer layer, the transparent conductive oxide, the electrodes, and an antireflection layer are deposited. Figure S1, Supporting Information, presents a cross‐sectional, annotated rendering of the complete solar cell architecture.

To the best of our knowledge, this paper is the first to present an inkjet printing‐based, scalable hybrid two‐step process to fabricate fully textured PSTs. Our process achieves performance approaching that of currently fabricated spin‐coating‐based hybrid‐deposited PSTs and enables comparable conformal growth on large textured silicon solar cells.^[^ ^13^, ^16^ ^]^ By combining hybrid two‐step perovskite deposition with the precise spatial control of inkjet printing, we demonstrate industrial manufacturing precision, enabling the applicability to state‐of‐the‐art edge isolation. **Figure** **1** illustrates the fabrication of a PST. First, the hole transport layer (HTL) is deposited onto the silicon solar cell (Figure 1a), followed by vapor‐phase deposition of the inorganic components, which constitute the first step in the perovskite deposition process (Figure 1b). Next, the organic precursor materials are inkjet‐printed, which constitutes the second step in the perovskite deposition process (Figure 1c). After perovskite formation, the passivation, electron transport layer (ETL), and front‐side electrode are deposited to finish the tandem solar cell. Finally, the antireflection coating is deposited to enhance the absorption (Figure 1d).

In the following, the results are presented in three stages. In the first part, we present the development of a high‐efficiency wide bandgap perovskite solar cell using hybrid two‐step inkjet printing. By combining this approach with an effective passivation strategy, we achieve a single‐junction device with a PCE of 19.8%. In the second part, we transfer and optimize this process for fully textured silicon bottom cells, culminating in a monolithic PST with a PCE of 27.4%. In the final part, we highlight the industrial relevance of the inkjet printing approach, demonstrating its high spatial deposition precision, enabling accurate edge isolation.

### 2.1 Development of High‐Efficiency Wide Bandgap Perovskite Solar Cells

One of the greatest challenges in realizing high‐quality two‐step processed perovskite thin films is ensuring accurate stoichiometry of the perovskite film. In contrast to one‐step processed thin films, small variations in layer thickness of the precursor materials deposited in the first or second step result in significant changes in the final composition. As previously reported by our group, insufficient organic deposition results in an inorganic layer atop the HTL, which parasitically absorbs light beyond the bandgap of lead iodide (PbI_2_), thereby reducing the short‐circuit current density (*J*_SC_).^[^ ^22^ ^]^ In contrast, excess deposition of organic precursor materials results in a decrease in open‐circuit voltage (*V*_OC_), potentially due to the formation of pinholes, cracks, and poor interfacial contact.^[^ ^22^, ^23^ ^]^ It should therefore be noted that finding the correct printing resolution to achieve stoichiometry and hence best performance is of utmost importance for achieving the following results.

**Figure 2:**
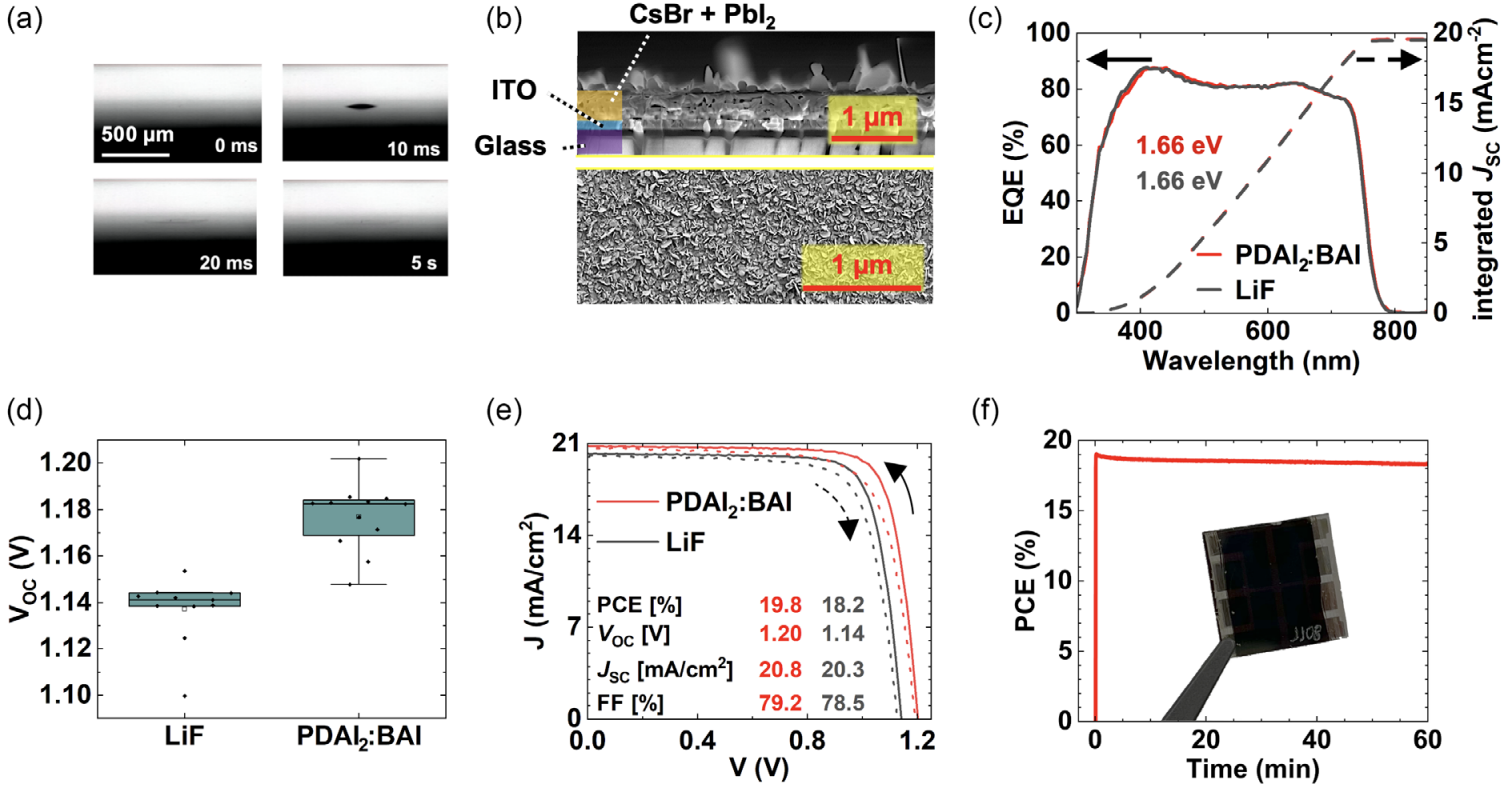
a) Time‐resolved microscopy images showing the absorption of a 50 pL droplet into the evaporated inorganic thin film. b) Cross‐sectional and surface SEM images of the respective inorganic thin film. The purple area corresponds to glass, the blue area corresponds to indium tin oxide, and the orange area corresponds to cesium bromide and lead iodide. c) EQE and integrated short‐circuit current density of the champion single‐junction perovskite solar cell. d) Distribution of the open‐circuit voltage for single‐junction perovskite solar cells fabricated with lithium fluoride compared to propane‐1,3‐diammonium iodide:n‐butylammonium iodide as surface passivation. e) Current density–voltage curves of the champion single‐junction perovskite solar cells fabricated with lithium fluoride compared to propane‐1,3‐diammonium iodide:n‐butylammonium iodide as surface passivation. f) Image of the fabricated single‐junction perovskite solar cell and 1 h stabilized maximum power point tracking of the champion single‐junction perovskite solar cell.

Pappenberger et al. demonstrate that wide bandgap PSCs fabricated using a two‐step process, incorporating bromide in both deposition steps, are advantageous. Furthermore, incorporating Cs into the perovskite thin film has been reported to enhance stability and performance while increasing reproducibility.^[^ ^24^, ^25^ ^]^ In the first step, one possibility to include both is to evaporate either cesium bromide (CsBr) or cesium chloride (CsCl) along with PbI_2_. This work uses both CsBr:PbI_2_ and CsCl:PbI_2_ thin films through evaporation, as reported in literature by Chin et al., who evaporated CsBr and PbI_2_.^[^ ^13^ ^]^ As presented by our group, hybrid two‐step inkjet printing necessitates porous inorganic thin films to allow sufficient absorption of the picoliter‐size droplets for good precursor intermixing. Furthermore, we showed that fast absorption times (<1s) combined with an introduced horizontal and vertical step size for inkjet printing are necessary to prevent large area drying effects like the coffee ring effect. The introduced step size increases the time interval between the deposition of neighboring droplets, allowing sufficient time for penetration to occur before droplet coalescence leads to the formation of a continuous wet film. Using time‐of‐flight secondary ion mass spectrometry, we found that nonporous inorganic thin films with droplet absorption times longer than 1 s exhibited low droplet penetration. In contrast, our group demonstrated that faster absorption, measurable using time‐resolved microscopy, correlates with enhanced intermixing and improved precursor distribution.^[^ ^22^ ^]^ Here, profilometry and time‐resolved microscopy images indicate sufficient porosity automatically occurring for the evaporated inorganic thin films consisting of CsBr and PbI_2_. Time‐resolved microscopy images reveal complete droplet absorption in the evaporated inorganic thin film within 20 ms (**Figure** **2**a). Profilometry of the picoliter‐sized droplet on the inorganic thin film compared to a similar droplet after drying on a planar surface reveals the same trend (Figure S2a, Supporting Information). While 24 nm CsBr and 240 nm PbI_2_ are evaporated, a height exceeding the expected 264 nm occurs (Figure 2b). Profilometry measurements across a scratch in the inorganic thin film support this observation, revealing nearly a twofold increase in thickness (Figure S2b, Supporting Information). This suggests a low density of the inorganic thin film, which can be attributed to porosity, beneficial for hybrid two‐step inkjet printing. Additionally, the droplet containing the organic precursor materials absorbed by the evaporated inorganic thin film exhibits spreading along its edge, indicating improved interdiffusion of the droplets and hence distribution of the organics (Figure S2c, Supporting Information). Lastly, X‐ray diffraction of the annealed perovskite thin film reveals a dominating 100‐perovskite peak and a weak PbI_2_ peak, indicating sufficient conversion (Figure S2d, Supporting Information). We conclude that the evaporated thin films exhibit sufficient picoliter droplet absorption for large area drying‐pattern free hybrid two‐step inkjet printing.

Building on the above‐reported recipe, highly efficient PSCs are fabricated using evaporated CsBr:PbI_2_ thin films. For inkjet printing of the organic precursor materials in the second deposition, we used a modified version of the formamidinium bromide (FABr) and FAI‐containing recipe. Instead of mixing FABr:FAI in a 1.5 ratio, we reduced the ratio to 1.2 and added 0.13 m methylammonium chloride (MACl) to maintain a wide bandgap and enhance crystallinity, as reported in several studies.^[^ ^26^, ^27^, ^28^ ^]^ To achieve stable droplet formation during inkjet printing, we selected a molarity of 0.59 m, composed of 0.21 m FAI, 0.25 m FABr, and 0.13 m MACl. While lower molarities can be used, they require higher DPI and thus result in longer inkjet printing times.^[^ ^22^ ^]^ In contrast, higher molarities are unsuitable because the ink tends to precipitate more quickly, leading to clogging of the inkjet printer nozzles (Figure S3, Supporting Information). Given that the amount of organics deposited permits complete conversion of the inorganic thin film, this recipe results in a nominal composition of Cs_0.16_FA_0.65_MA_0.19_Pb(I_0.77_Br_0.17_Cl_0.06_)_3_. External quantum efficiency (EQE) measurements on the champion PSCs show a bandgap of 1.66 eV (Figure 2c). PSCs produced by this recipe and using 1 nm of lithium fluoride (LiF) as surface pssivation achieved a median PCE of 17.8%, with a median *V*_OC_ of 1.14 V, a median *J*_SC_ of 20.0 mA cm^−^^2^, and a median FF of 77.8% (Figure S4a‐c, Supporting Information and Figure 2d). In order to improve the C_60_‐perovskite interface, we applied a propane‐1,3‐diammonium iodide:n‐butylammonium iodide (PDAI_2_:BAI) surface passivation. PDAI_2_‐based passivation techniques are widely used in perovskite solar cell research, with Liu et al. mentioning PDAI_2_:BAI first.^[^ ^29^, ^30^, ^31^, ^32^ ^]^ Pappenberger et al. demonstrate that this passivation in a 1:1 mass ratio is well‐suited for two‐step fabricated PSCs, offering improved *V*_OC_ and enhanced long‐term stability. Furthermore, the dual passivation strategy outperformed the passivation capabilities of either component used individually.^[^ ^33^ ^]^ According to literature, this improvement is attributed to suppressed nonradiative recombination, particularly through effective passivation of grain boundary defects, as well as favorable band alignment at the treated interfaces, which enhances carrier extraction.^[^ ^32^, ^33^ ^]^ To assess how surface passivation influences the device performance by reducing trap‐state density, we perform photoluminescence quantum yield (PLQY) measurements. Using PLQY, the implied *V*_OC_ (i*V*_OC_) can be determined.^[^ ^34^, ^35^ ^]^ If exposed to one sun excitation, i*V*_OC_ values with and without C_60_ follow a similar trend to the measured *V*_OC_ results. Without C_60_, perovskite thin films passivated with LiF exhibit a median i*V*_OC_ of 1.24 V, while replacing LiF with PDAI_2_:BAI increases i*V*_OC_ by 10 meV (Figure S4d, Supporting Information)  As reported in literature, the deposition of C_60_ leads to a decrease in PLQY and, consequently, in i*V*_OC_, which can be attributed to significant interfacial recombination at the perovskite‐C_60_ interface.^[^ ^36^, ^37^ ^]^ Remarkably, compared to LiF‐passivation, the i*V*_OC_ still remains 10 meV higher for PDAI_2_:BAI‐passivated perovskite thin films, hinting toward an improved perovskite‐C_60_ interface (Figure S4d, Supporting Information). These findings align with the slight improvements in FF and *V*_OC_ observed in single‐junction PSC measurements (Figure 2d and Figure S4c, Supporting Information). In this work, PSCs passivated using PDAI_2_:BAI resulted in a median increase of 0.04 V in *V*_OC_, 0.23 mAcm^−2^ in *J*_SC_, and 0.63% in FF (Figure 2d and Figure S4b,c, Supporting Information). Overall, this results in a median increase in PCE of 0.65% (Figure S4a, Supporting Information), to a median PCE of 18.43%, and a champion device showing a PCE of 19.84% (Figure S4a, Supporting Information and Figure 2e). To the best of our knowledge, these are the most efficient wide bandgap PSCs reported using an inkjet printing step. Furthermore, the champion PSC demonstrates promising MPP stability, remaining stable for one hour under standard test conditions despite the perovskite being partially fabricated under ambient conditions (Figure 2f).

To address the scalability of our hybrid two‐step process, we utilize a square base substrate with an area of 40.96 cm^2^ as the platform for fabricating small PSCs. The substrate is patterned with indium tin oxide (ITO) electrodes to accommodate 64 uniformly distributed PSCs, each with an active area of 10.5 mm^2^. Following the evaporation of the inorganic layer (Figure S5a, Supporting Information), the printing of the organic precursor material, and subsequent annealing to form the final perovskite structure (Figure S5b, Supporting Information), a visually uniform thin film is obtained across the entire substrate. After completing the processing on a large area, the base substrate is diced into 16 smaller substrates (16 mm^2^), each containing four of the 10.5 mm^2^ PSCs. With a standard deviation of merely 2.88% in PCE (median PCE 16.44%), we consider the electrical performance across the substrate area to be highly uniform (Figure S5c, Supporting Information).

### 2.2 Highly Efficient Fully Textured Perovskite/Silicon Tandem Solar Cells

**Figure 3:**
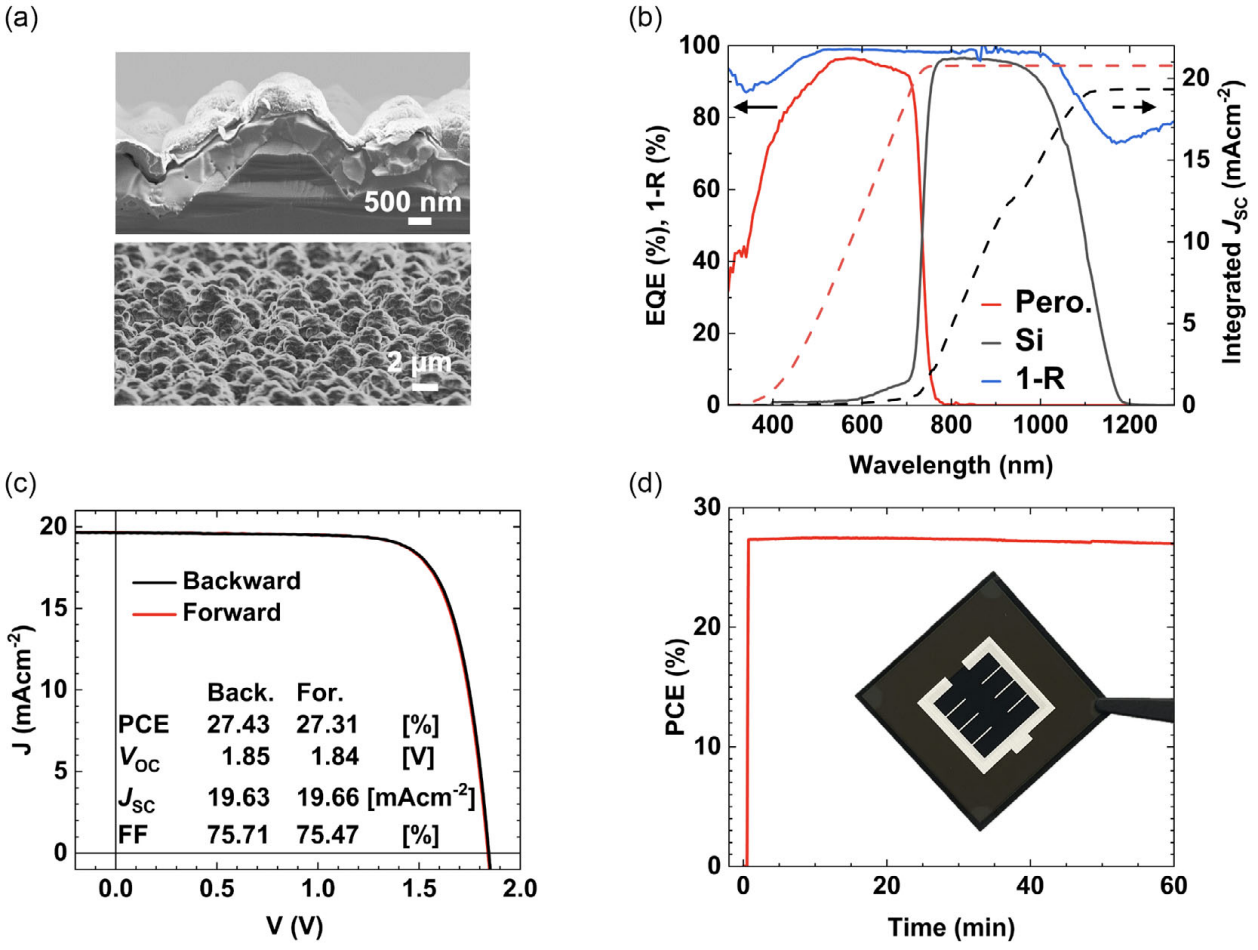
a) Cross‐sectional and surface SEM images of a fully textured perovskite/silicon tandem solar cell. b) EQE, 1‐reflectance, and integrated short‐circuit current densities for both the perovskite and silicon solar cells. c) Backward and forward current density‐voltage curves of the best‐performing perovskite/silicon tandem solar cell. d) Image of the fabricated perovskite/silicon tandem solar cell and 1 h stabilized maximum power point tracking data.

Hybrid two‐step deposition is applicable to silicon‐based bottom solar cells, enabling the fabrication of highly efficient PSTs. Our previous work demonstrates conformal perovskite deposition due to the combination of evaporation and inkjet printing. This enables the fabrication of PSCs on industry‐relevant silicon bottom solar cells with featured textures (>3 μm) and saw damages exceeding the perovskite thin film's height (<1 μm).^[^ ^22^ ^]^ Heterojunction silicon solar cells from *Meyer Burger Technology AG* with 3–5 μm textured surfaces are used as a proof of concept. To achieve complete coverage for the conformally grown perovskite thin film, we increased the nominal evaporation thickness of the inorganics from 24 nm CsBr + 240 nm PbI_2_ to 50 nm CsBr + 500 nm PbI_2_. Due to the increased surface area of the textures, a thickness similar to that of the perovskite fabricated on planar single‐junction solar cells is achieved (**Figure** **3**a and Figure S6, Supporting Information). To maintain the same inorganics‐to‐organics ratio despite the overall increase in the inorganic material, the printing resolution is increased simultaneously. A DPI of 1300 is required to convert the 550 nm inorganic thin film and hence enable the highest PCEs. In addition to the adaptation to achieve comparable perovskite thickness, the bandgap of the perovskite top solar cell was slightly increased by 30 meV to improve the matching of the currents produced in the top and bottom solar cells. Compared to the original recipe, the CsBr layer was replaced by an equally thick CsCl layer (Figure S7, Supporting Information). Due to its 20% lower molar mass and 10% lower density, the incorporation of CsCl results in a 3% increase in the ratio of bandgap‐enhancing Cs cations relative to MA and FA cations. Additionally, the proportion of bandgap‐enhancing Cl and Br halides relative to I increases by 2%. Ultimately, a nominal composition of Cs_0.18_FA_0.63_MA_0.19_Pb(I_0.76_Br_0.12_Cl_0.12_)_3_ results. Using EQE measurements, an integrated *J*_SC_ of 19.35 mAcm^−2^ for the silicon bottom solar cell and 20.78 mAcm^−2^ for the perovskite top solar cell occurs (Figure 3b). Exchanging CsBr with CsCl, as well as depositing the inorganic precursor materials onto textured silicon solar cells instead of planar ITO‐glass substrates, did not affect the formation of a rough, low‐density inorganic thin film (Figure S8, Supporting Information). Finally, a highly efficient tandem solar cell with a PCE of 27.43% is fabricated (Figure 3c). It should be noted that the mean PCE is 25.6% (Figure S9, Supporting Information). The conformal growth of the perovskite top solar cell preserves the textures of the silicon bottom solar cell, resulting in excellent light management and 1‐reflectance (absorptance) values approaching 100% (Figure 3a,b). By applying the same surface passivation strategy used for the single‐junction solar cells, a *J*_SC_ of 19.66 mAcm^−2^, a *V*_OC_ of 1.85 V, and an FF exceeding 75% are measured for the best‐performing device (Figure 3c). The hysteresis in the PCE is 0.12%, which indicates a high‐quality perovskite and good interfaces. MPP‐tracking for over 1 h and in ambient atmosphere shows a similar stable output as observed with the single‐junction solar cells: The tandem solar cell maintained a PCE of over 27% for one hour (Figure 3d). This indicates low defect densities and good crystallinity.^[^ ^38^ ^]^ Long‐term MPP‐tracking in ambient atmosphere and over the course of 500 h decreases PCE to 22% within 24 h, remaining stable for 500 h afterward (Figure S10, Supporting Information). We assume that adequate perovskite encapsulation can mitigate degradation, as exposure to humidity may partially decompose the perovskite, increasing the concentration of free ions. Thiesbrummel et al. demonstrated that mobile ion‐induced field screening leads to light‐induced degradation, primarily reflected in reduced *J*_SC_ and FF, an effect which we also observe, further supporting this assumption.^[^ ^39^ ^]^

### 2.3 Fulfilling Edge Isolation Requirements Using Inkjet Printing

One often overlooked challenge in upscaling perovskite/silicon tandem photovoltaics is the need for excellent processing of the perovskite top cell across the entire silicon wafer, including the edges. Turbulence in the precursor ink or flow‐induced effects during drying can significantly impact the film homogeneity near the wafer edges.^[^ ^2^, ^40^ ^]^ In heterojunction silicon solar cells, the acceptable tolerance for edge‐related inhomogeneities is defined by edge isolation, typically around 500 μm. In passivated emitter rear cell and tunnel oxide passivated contact (TOPCon) solar cells, edge isolation is typically performed using either laser‐based or wet chemical methods, allowing isolation to reach the very edge of the wafer.^[^ ^41^ ^]^ Here, we demonstrate that our proposed hybrid processing route, which employs inkjet‐printed organic precursors, enables the fabrication of high‐performance devices with uniform quality extending all the way to the wafer edges.

**Figure 4:**
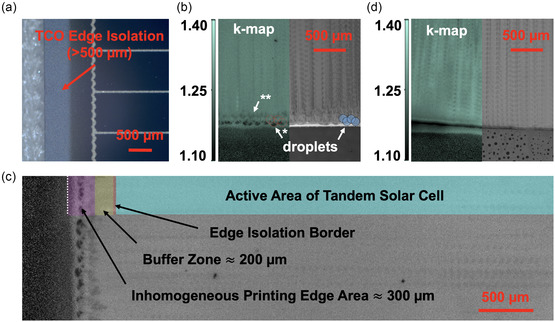
a) Microscopy image of the edge isolation region on an industrial heterojunction silicon solar cell. k‐maps and microscope images of hybrid two‐step inkjet‐printed perovskite thin films: b) one printed up to the edge of the substrate and d) one printed over the edge. In (b), * indicates the lower k‐values occurring due to a missing neighboring droplet, and ** indicates the lower k‐values occurring due to print head acceleration limitations. c) k‐map of the perovskite thin film on the outer millimeters of the substrate, highlighting the high‐quality perovskite formation region compared to the area required for edge isolation.

A widely adopted industrial silicon doping method is the thermal diffusion process, enabling single‐sided doping.^[^ ^42^ ^]^ In this technique, silicon wafers are exposed to a dopant source within a diffusion chamber. However, due to slight surface irregularities or small gaps at the wafer edges, unintended doping of the reverse side can occur. To mitigate the resulting risks of short circuits and recombination losses, an edge isolation step is commonly employed.^[^ ^21^, ^43^ ^]^ Using crystalline silicon, laser‐based methods are among the most precise edge isolation techniques, achieving laser trenches narrower than 50 μm (Figure S11a, Supporting Information).^[^ ^44^ ^]^ While research studies have demonstrated edge isolation distances as small as 200–300 μm, industrial processes employ larger isolation distances. Wet chemical edge isolation is the predominant method for PERC and TOPCon silicon solar cells, enabling complete removal of the rear‐side emitter through a single‐sided etching step. This approach permits the active use of the entire wafer area, right up to its edge.^[^ ^41^ ^]^ Although modern silicon solar cell technologies, such as heterojunction solar cells, deposit doped amorphous silicon using methods like chemical vapor deposition, the solar cells’ edges still require electrical isolation to prevent edge‐induced short‐circuits. Industrially, this is commonly achieved by applying transparent conductive oxides using masking techniques.^[^ ^45^, ^46^ ^]^ Consequently, the solar cell's active area begins several hundred micrometers away from the silicon solar cell edge (**Figure** **4**a).

In conclusion, a precise spatial control of perovskite deposition is necessary to meet the demands of industrial edge isolation. Here, we demonstrate that inkjet printing, as the second deposition step, enables precise perovskite fabrication up to 300 μm of the silicon bottom cell's edge without material losses, extending to its very edge with minimal material losses. As a result, no material loss would occur during the inkjet printing step on laser‐edge‐isolated or heterojunction silicon solar cells, while only minimal loss is expected for silicon solar cells using wet chemical edge isolation.

First, we investigate how close printing to an edge without exceeding it is possible. Assuming spherical droplets, the sharpness of a printed edge is limited to half the droplet's radius, as the next droplet necessary for forming a uniform perovskite thin film is missing at the edge. Consequently, smaller droplet volumes enable more precise printing closer to an edge. However, higher printing resolution and increased printing time are required to print the same volume using printheads with lower droplet volumes. This work employs the Sapphire QS‐256/10 AAA printhead from SÜSS, with a nozzle volume of 10 pL. Assuming a 90° contact angle and 100% nozzle volume ejection, the calculated droplet surface radius is 168.4 μm (Figure S11b,c, Supporting Information). A microscope image of an exemplary droplet printed shows that this calculation approximately matches the visible surface droplet radius of ≈120 μm (Figure S11b, Supporting Information). Furthermore, k‐maps reveal no major change in the perovskite's optoelectronic properties up to a distance equal to the droplet radius from the solar cell's edge, indicating uniform perovskite quality (Figure 4b, see *). Thickness measurements of the perovskite across the edge reveal a decrease only within ≈150 μm (≈half the droplet radius) from the edge (Figure S12a, Supporting Information). This indicates that the dark, half‐circle‐shaped spots observed in the k‐maps near the solar cell's edge result from improper perovskite composition, likely due to a lack of organics. It should be noted that an additional edge‐close 300 μm‐broad region with lower k‐values is observed. This is attributed to the printhead acceleration process and can be mitigated using an inkjet printer with advanced settings. (Figure 4b, see **). We conclude that the k‐value reduction at the perovskite edge, driven by inkjet printing, is primarily associated with the edge‐close half‐droplets and the printhead's acceleration at the outermost edge. Both phenomena occur within a spatial distance smaller than required for laser edge isolation or common edge isolation for heterojunction silicon solar cells. Figure 4c illustrates the perovskite formation region near a silicon solar cell's edge, demonstrating that edge isolation with high‐quality perovskite in the silicon solar cell's active area is achievable.

Printing beyond the solar cell's edge is an alternative method to achieve a uniform perovskite thin film up to the very edge of a solar cell, as required for wet chemical edge‐isolated silicon solar cells. However, this approach has the drawback of slight material yield losses due to the organic material being printed beyond the edge of the solar cell. Since inkjet printing enables precise deposition of picoliter droplets, it is possible to print exactly one additional droplet row beyond the solar cell's edge. For the 10 pL printhead used in this work, taking the M10 wafer size (182 × 182 mm^2^) as an example, an area of 182.34 × 182.34 mm^2^ must be printed, thereby maintaining a material yield of over 99.6%. Using this approach, a k‐map of the perovskite thin film shows no major change in the perovskite's optoelectronic properties up to the very edge of the solar cell, indicating consistent perovskite quality (Figure 4d).

In addition to ensuring uniform perovskite formation along the edge of a solar cell, inkjet printing is capable of free‐form deposition, enabling the printing of arbitrary shapes (Figure S12b,c, Supporting Information). This capability makes inkjet printing an even more promising candidate for hybrid two‐step perovskite deposition, since industrially relevant silicon solar cells, which are commonly fabricated on pseudo‐square wafers, can be printed along the wafer edge, even if the corners are truncated.

## 3 Conclusion

PSTs are among the most promising candidates for surpassing the efficiency limits of current photovoltaic technologies. In this study, we present a scalable hybrid two‐step deposition method that combines the evaporation of all inorganic precursor materials with an inkjet printing step for depositing the organic precursors. This approach facilitates excellent light management and enables the fabrication of highly efficient PSTs, achieving a champion device power conversion efficiency (PCE) exceeding 27.4% during one hour of maximum power point tracking in an ambient atmosphere. By using CsCl in place of CsBr during the evaporation process, we achieve an increased bandgap, while the incorporation of PDAI_2_:BAI for surface passivation leads to enhanced perovskite performance. The conformal growth inherent to the evaporation process, combined with the absorption of organic precursors into the inorganic thin film during the second deposition step, enables conformal perovskite deposition. Beyond demonstrating a record‐high PCE for a scalable‐fabricated, fully textured PST, we validate the applicability of this deposition method within existing silicon solar cell manufacturing processes. One widely overlooked challenge in upscaling perovskite/silicon tandem photovoltaics is to meet the requirements of precise spatial deposition for edge isolation. In silicon solar cell production, edge isolation is necessary to electrically isolate the solar cell's perimeter. Our hybrid two‐step inkjet printing method can deposit high‐quality perovskite layers up to the edge of the silicon bottom cell, ensuring superior perovskite formation on the active area of isolated tandem devices. Further optimization of the solar cell architecture using this method could enable scalable fabrication of PSTs with efficiencies exceeding 30%. Due to the scalability of both the evaporation and inkjet printing processes, there is no fundamental limitation to applying these high‐performance perovskite thin films to arbitrarily large areas, making this approach highly suitable for industrial‐scale photovoltaic production.

## 4 Experimental Section

4.1

4.1.1

##### PSC Fabrication

To fabricate the single‐junction perovskite solar cells (PSCs) presented in this study, structured ITO‐coated glass substrates (120 nm, 15 Ω) from Luminescence Technology Corp (Lumtec) were used. The substrates were sequentially cleaned in acetone (15 min) and isopropyl alcohol (IPA) for 15 min using an ultrasonic bath. After cleaning, they were dried with a nitrogen gun and subjected to oxygen plasma treatment (100% O_2_, 15 min). For HTL deposition, 2PACz (0.5 mg mL^−1^, TCI) dissolved in ethanol (>99.8%, VWR Chemicals) was spin‐coated onto the substrates. The solution was prepared one week before use and sonicated in an ultrasonic bath for 60 min immediately after preparation and prior to deposition. A total of 80 μL was spin‐coated in a nitrogen‐filled glovebox at 3000 rpm with an acceleration of 1000 rpm s^−^^1^ for 30 s. Following spin‐coating, the substrates were annealed at 100 °C for 10 min on a hotplate. To enhance uniformity, an additional ethanol rinsing step was performed by spin‐coating 80 μL of ethanol under identical spin‐coating conditions, followed by a second annealing step at 100 °C for 10 min. After HTL deposition, the substrates were transferred to an M. Braun Intergas‐Systeme GmbH evaporation system to deposit the inorganic perovskite precursors. CsBr (24 nm, 0.1 Å s^−1^, TCI) and PbI_2_ (240 nm, 1 Å s^−1^, TCI) were evaporated. The organic cation precursor solution contained 35.27 mg mL^−^^1^ FAI (GreatCell Solar), 30.64 mg mL^−^^1^ FABr (GreatCell Solar), and 9 mg mL^−^^1^ MACl (GreatCell Solar) dissolved in IPA (99.5%, Sigma–Aldrich), with dissolution facilitated by vortex shaking. The resulting nominal composition was Cs_0.16_FA_0.65_MA_0.19_Pb(I_0.77_Br_0.17_Cl_0.06_)_3_. To determine the exact perovskite stoichiometry, it was assumed that the material followed the ABX_3_ crystal structure, with PbI_2_ as the exclusive B‐site cation. The moles corresponding to a complete perovskite conversion were calculated, given the known density of pristine PbI_2_ (6.16 g cm^−3^) and a deposited film thickness of 240 nm. Using the mass ratios and molar masses of the organic salts, the molar ratios of the A‐site cations (FA, MA, Cs) and the X‐site anions (I, Br, Cl) were derived. Inkjet printing was performed using a Pixdro LP50 inkjet printer from SÜSS, equipped with a Sapphire QS‐256/10 AAA printhead (10 pL, SÜSS). Although the technical data sheet specifies a suitable viscosity range of 8–20 cP for this printhead, our ink demonstrated stable jetting for at least 8 h despite having a room‐temperature (21.8 °C) viscosity of only 3.54 cP. The printhead was operated using a waveform with the following parameters: width = 9 μs, space = 5.7 μs, level = 45.88 V, rise/fall = 64.7 V μs^−1^, resulting in an average droplet size of 10 pL. It should be noted that the droplet size is determined by the inkjet printer's in situ droplet analysis tool, which calculates droplet volume based on the detected diameter. This detection is influenced by the background illumination. Variations in background light intensity can lead to a fluctuation of ≈ +/−2 pL in the calculated droplet volume. Given that the nominal nozzle volume is 10 pL, we consider this to be the most accurate estimate of the actual droplet volume. The firing frequency was set to 2 kHz, which, combined with a resolution of 900 DPI, resulted in a print speed of 56.4 mm s^−1^. Lastly, the printhead height was set to maintain 100 μm above the substrate during deposition. Prior to printing, the inorganic thin films were removed from the nitrogen atmosphere. Both the vertical (printhead travel direction) and horizontal (perpendicular to printhead travel) step sizes were set to 8. The printhead remained in its native orientation throughout the printing process. Across all fabrication batches, optimal device performance was achieved with an inkjet printing resolution of around 900 DPI for the perovskite thin film. Following organic precursor deposition, the PSCs were annealed under ambient conditions (20 °C, 40%–45% relative humidity) on a hotplate at 150 °C for 15 min before being returned to a nitrogen‐filled glovebox. Subsequently, a passivation layer (1 nm lithium fluoride, 0.2 Å s^−1^, ChemPur), an ETL (20 nm C_60_, 0.2 Å s^−^^1^, Sigma–Aldrich), and a buffer layer (3.5 nm Bathocuproine (BCP), 0.2 Å s^−1^, Lumtec) were deposited one after another in an Angstrom Engineering thermal evaporator. For the champion devices, LiF was replaced with PDAI_2_:BAI surface passivation. A solution of 0.3 mg mL^−^^1^ PDAI_2_ (GreatCell Solar) in IPA was prepared by sonicating it for 24 h. Separately, a 0.3 mg mL^−1^ BAI (GreatCell Solar) solution in IPA was prepared using vortex shaking for 5 min. The two solutions were then combined and mixed for 5 min using a vortex shaker. Finally, the passivation solution was spin‐coated onto the perovskite at 4000 rpm with an acceleration of 1000 rpm s^−1^ for 30 s. The IPA was evaporated through annealing on a 100 °C hotplate for 5 min. Then, as with the LiF‐based devices, C_60_ and BCP were subsequently deposited. After evaporation, the ITO contacts were re‐exposed by mechanically removing excess material using a scalpel. Finally, 100 nm of silver was thermally evaporated at a deposition rate of 0.5 Å s^−1^ through a shadow mask using a VacTec COAT340 evaporator. The ITO front contacts and the silver contact layout enabled the fabrication of four PSCs (10.5 mm^2^ each) per substrate.

##### PSTs Fabrication

Heterojunction silicon bottom solar cells from *Meyer Burger Technology AG* were used to fabricate the PSTs presented in this study. Silicon bottom solar cells were fabricated as reported.^[^ ^13^ ^]^ On the front side, hydrogenated intrinsic and phosphorus‐doped amorphous silicon (a‐Si:H(i) and a‐Si:H(n)) depositions were followed by a phosphorus‐doped nanocrystalline silicon (nc‐Si:H(n)) deposition. An additional layer of boron‐doped nc‐Si:H(p) was deposited to form a tunnel junction. On the rear side, the deposition of a‐Si:H(i) was followed by an a‐Si:H(p) layer. Before processing, the solar cells were exposed to light soaking under an AM1.5 G solar simulator for 15 min. Subsequently, a dynamic spin‐coating cleaning routine was performed using acetone and IPA. The cleaning procedure was conducted at 2500 rpm with an initial acceleration of 1000 rpm s^−^^1^ for 30 s. After cleaning, the silicon bottom solar cells were dried on a 100 °C hotplate for 10 min. Following the cleaning process, the samples were transferred to a nitrogen‐filled glovebox. Next, the HTL, 4‐(3,6‐diphenyl‐9H‐carbazol‐9‐yl)butyl)phosphonic acid (0.5 mg mL^−1^) dissolved in methanol was deposited by spin‐coating 150 μL. Prior to deposition, the solution was sonicated in an ultrasonic bath for 3 h to ensure complete dissolution. The spin‐coating process was performed at 3000 rpm with an acceleration of 1000 rpm s^−^^1^ for 30 s. Following deposition, the methanol was removed by annealing the substrates at 100 °C for 10 min. In some cases, wetting of the HTL was improved using inorganic nanoparticles. Next, the samples were transferred to an M. Braun Intergas‐Systeme GmbH evaporation system to deposit the inorganic perovskite precursors. Here, CsCl (50 nm, 0.1 Å s^−^^1^) and PbI_2_ (500 nm, 1 Å s^−^^1^) were evaporated under high vacuum. The standard organic cation precursor ink used in this work contained 35.27 mg mL^−^^1^ FAI, 30.64 mg mL^−^^1^ FABr, and 9 mg mL^−^^1^ MACl dissolved in IPA. The solution was homogenized using a vortex shaker. This recipe resulted in the perovskite composition Cs_0.18_FA_0.63_MA_0.19_Pb(I_0.76_Br_0.12_Cl_0.12_)_3_, which was determined using the same methodology as described for the single‐junction devices. The inkjet printing steps followed the same procedure as described for single‐junction devices, except for the printing resolution, which was increased to 1300 DPI to accommodate the increased inorganic film thickness. Following perovskite deposition, the devices were transferred back into a nitrogen‐filled glovebox. PDAI2:BAI surface passivation was applied as described for the single‐junction PSCs, except that only 0.1 mg mL^−^^1^ of each material was used. For the ETL, 20 nm of C_60_ was thermally evaporated at a deposition rate of 0.2 Å s^−^^1^ using an Angstrom Engineering thermal evaporator. A 20 nm SnO_2_ thin film is deposited using reactive atomic layer deposition in a GEMStar XT system from Arradiance, with tetrakis(dimethylamino)tin(IV) TDMASn and water as precursors. The TDMASn pulse lasts 1.6 s, followed by a 12 s purge, while the water pulse duration is 0.1 s with a 16 s purge. High‐purity nitrogen (99.999%) is used as the carrier and purge gas. The line flow rates are set to 120 sccm for TDMASn and 150 sccm for water. To ensure thermal stability, the TDMASn source container is preheated at 70 °C for one hour. A 45 nm transparent indium zinc oxide electrode was then sputtered at 1 mTorr using 190 W power with pure Ar and O_2_, employing a sputtering system from Kurt J. Lesker. To define the active area, a mask with a 1.04 cm^2^ centered opening was used. Silver (Ag) or gold (Au) was thermally evaporated as the front‐side electrode material, with a final thickness of 600 nm. To improve charge extraction, six evenly distributed 150 μm‐wide metal fingers extended partially over the active area. Finally, to minimize reflection losses, a 125 nm MgF_2_ antireflection coating was thermally evaporated onto the electrode. The thicknesses of all layers deposited on textured surfaces are reported as nominal values, as determined by readings from the quartz crystal microbalance. It should be noted that the actual layer thicknesses are lower due to the increased surface area caused by the texture.

##### J–V Measurement

For measuring the single‐junction PSCs characteristics, a xenon‐lamp‐based solar simulator (Newport Oriel Sol3A) with an AM1.5 G spectrum (1000 Wm^−2^) was calibrated using a certified silicon solar cell (Newport) equipped with a KG5 band‐pass filter. Subsequently, the PSCs were measured in backward and forward directions with a step size of 10 mV. All cells were measured from 0.2 to 1.3 V with a scanning rate of 0.6 V s^−1^ using a Keithley 2401 source measurement unit. During the measurement, the temperature of the PSCs was held at 25 °C with a microcontroller‐adjusted Peltier element. The measurements were performed in a nitrogen glove box. A pre‐calibrated light‐emitting diodes (LED)‐based solar simulator (Sinus‐70 Advanced) from WAVELABS Solar Metrology Systems GmbH was used to measure the PSTs. PSTs were measured in ambient atmosphere and from 0.2 to 2.0 V with a scanning rate of 0.6 V s^−1^ using a Keithley 2400 source measurement unit. For long‐term MPP tracking, a solar simulator from PURI Materials (PR‐LEDSUN‐22R) in an ambient atmosphere was used. For all cells, MPP was tracked by using a perturb‐and‐observe method. For PSTs, the illuminated area during measurement was fixed to 1 cm^2^ using a mask.

##### EQE Measurement

The EQEs were measured using a PVE300 PV QE system (Bentham EQE system). For calibration, a silicon and a germanium reference solar cell were used. All single‐junction measurements were done using a 0.74 mm^2^ illumination spot, while tandem solar cells were measured using a 1.5 mm^2^ illumination spot. For all EQE measurements, a chopping frequency between 560–590 Hz and an integration time of 500 ms (for single‐junction PSCs) and 750 ms (for PSTs) was used. To measure the EQEs of the sub‐cells in the PST, bias light was used, which saturated either the silicon or the perovskite solar cell. The measurement step size was 2–5 nm. The measurements were performed in a nitrogen glove box.

##### Reflectance Measurement

The perovskite thin films reflectance spectra were measured using a PerkinElmer Lambda1050 spectrophotometry setup equipped with a double monochromator and a modulated source. A chopper frequency of 46 Hz was applied.

##### Time‐Resolved Microscopy Images

Time‐resolved microscopy images were recorded using an optical contact angle and drop contour analyzer (DataPhysics Instruments GmbH, OCA 200) applied with the PicoDrop kit. Hereby, cartridges jetting 50 pL droplets were used.

##### PLQY

PLQY measurements were performed using a LuQY Pro system from QYB Quantum Yield Berlin GmbH. The device's software calculated the resulting i*V*_OC_ values, factoring in the EQE of the perovskite thin films. To conduct the measurements, the samples were placed inside an integrating sphere, where a green laser (λ = 532 nm) was introduced through a small entrance port.

##### Scanning Electron Microscopy (SEM)

SEM analysis was conducted in an SEM (Zeiss LEO1530) with an in‐lens detector and an aperture size of 20–30 μm. The applied acceleration voltages for surface and cross‐sectional analysis ranged between 3 and 5 kV.

##### Microscopy

Microscope images were taken with a Zeiss Axioplan 2 imaging.

##### Profilometry

The thickness of the perovskite thin films was measured using a Bruker Dektak XT profilometer.

##### Viscosity

Viscosity measurements were performed using a setup from Anton Paar. The measurements were carried out with a ViscoQC 300‐L rotational viscometer equipped with a PTD 100 temperature control unit and a CP40 cone‐plate spindle. All measurements were conducted at room temperature (21.8 +/−0.1 °C) with a sample volume of 0.5 mL. To ensure optimal measurement accuracy, the rotational speed was adjusted such that the relative torque exceeded 90% of the instrument's maximum, as recommended by the manufacturer. The rotational speed resulted in 85 rpm. After loading the ink, measurements were initiated and monitored until a stable reading was achieved, typically within 10–15 s. The estimated measurement uncertainty was +/−1%.

##### Photoluminescence and k‐Mapping

To perform photoluminescence and k‐mapping, two 467 nm LED bars (LDL2‐170/30‐BL2, Creating Customer Satisfation Inc.) powered by a power control unit (PD3‐5024‐4‐EI, Creating Customer Satisfation Inc.) were used to excite the sample in a 45° symmetrical alignment. The image was recorded by a sCMOS camera (CS2100M‐USB – Quantalux 2.1 MP Monochrome sCMOS Camera, Thorlabs) with a macro zoom lens (Zoom 7000, Navitar) and an exposure time of 50 ms. A 695 nm absorptive long‐pass filter (092 MRCIR – M55.0 × 0.75, Schneider–Kreuznach) was attached to the lens to eliminate the detection of excitation light. Spatially‐resolved photoluminescence (*I*_*PL*_) was recorded with an excitation intensity (*I*_exc_) range of 0.005–0.2 suns, and a background correction at zero excitation was performed. The parameter k is extracted as the exponent of the power‐law model fit *I*_PL_≈*I*_exc_^k^, which is applied pixel‐wise to the excitation intensity‐dependent photoluminescence images.^[^ ^47^ ^]^ This parameter k, also referred to as the “optical ideality factor”, gives insight into the dominating recombination processes and, hence, the thin film quality.

##### Statistical Analysis

Boxplots use symbols to visually represent key statistical insights. The colored blocks represent the interquartile range (IQR), which spans from the 25th to the 75th percentile of the data. A vertical line extends 1.5 times the IQR, aiding in identifying potential outliers. The horizontal line denotes the median, while the hollow square signifies the mean. Data points and outliers falling outside the expected range are depicted as rhombuses.

## Supporting Information

Supporting Information is available from the Wiley Online Library or from the author.

## Conflict of Interest

The authors declare no conflict of interest.

## Author Contributions

**Raphael Pesch**: conceptualization (equal); data curation (lead); formal analysis (lead); investigation (lead); methodology (lead); visualization (lead); writing—original draft (lead). **Julian Petry**: data curation (supporting); formal analysis (supporting); investigation (supporting); writing—review and editing (supporting). **Julian Petermann**: data curation (supporting); formal analysis (supporting); visualization (supporting); writing—review and editing (supporting). **Ronja Pappenberger**: formal analysis (supporting); investigation (supporting); writing—review and editing (supporting). **Theresa Kuechle**: formal analysis (supporting); writing—review and editing (supporting). **Lena Paula Rothbauer**: investigation (supporting). **Johannes Schenck**: investigation (supporting). **Lingyi Fang**: data curation (supporting); formal analysis (supporting); writing—review and editing (supporting). **Xuzheng Liu**: data curation (supporting); formal analysis (supporting); methodology (supporting); writing—review and editing (supporting). **Saeid Rafizadeh**: methodology (supporting); resources (supporting); writing—review and editing (supporting). **Bahram Abdollahi Nejand**: formal analysis (supporting); investigation (supporting); methodology (supporting); resources (supporting); validation (supporting); writing—review and editing (supporting). **Johannes Sutter**: conceptualization (equal); formal analysis (supporting); funding acquisition (supporting); investigation (supporting); project administration (supporting); supervision (equal); validation (supporting); writing—review and editing (equal). **Ulrich Lemmer**: funding acquisition (supporting); resources (supporting); supervision (supporting); writing—review and editing (supporting). **Ulrich Wilhelm Paetzold**: conceptualization (equal); formal analysis (supporting); funding acquisition (lead); project administration (lead); resources (supporting); supervision (equal); validation (supporting); writing—original draft (supporting); writing—review and editing (lead).

## Supporting information

Supplementary Material

## Data Availability

The data that support the findings of this study are available from the corresponding author upon reasonable request.
