# Supplementary material for: Efficient Perovskite/Silicon Tandem Solar Cells Using Hybrid Two‐Step Inkjet Printing with Edge Isolation Precision
Source: Small Sci. 2025 Sep 23;5(11):2500362. doi: 10.1002/smsc.202500362 (PMC12622546; doi:10.1002/smsc.202500362)
Supplement: Supplementary file 1 — Supplementary Material [file SMSC-5-2500362-s001.pdf]

Supporting Information

Efficient Perovskite/Silicon Tandem Solar Cells Using Hybrid Two-Step Inkjet Printing With Edge Isolation Precision

Authors & Affiliation
Raphael Pesch*, Julian Petry, Julian Petermann, Ronja Pappenberger, Theresa Kuechle, Johannes Schenck, Lena P. Rothbauer, Lingyi Fang, Xuzheng Liu, Saeid Rafizadeh, Bahram Abdollahi Nejand, Johannes Sutter, Ulrich Lemmer, and Ulrich W. Paetzold*

R. Pesch, J. Petry, J. Petermann, R. Pappenberger, Dr. L. Fang, X. Liu, Dr. J. Sutter, Prof. Dr. U. Lemmer, Prof. Dr. U. W. Paetzold,
Institute of Microstructure Technology (IMT), Karlsruhe Institute of Technology (KIT), Hermann-von- Helmholtz-Platz 1, 76344 Eggenstein-Leopoldshafen, Germany

Email: raphael.pesch@kit.edu, ulrich.paetzold@kit.edu

R. Pesch, J. Petermann, R. Pappenberger, T. Kuechle, J. Schenck, L. P. Rothbauer, Dr. J. Sutter, Prof. Dr. U. Lemmer, Prof. Dr. U. W. Paetzold,
Light Technology Institute (LTI), Karlsruhe Institute of Technology (KIT), Engesserstrasse 13, 76131 Karlsruhe, Germany

Dr. S. Rafizadeh, Dr. B. Abdollahi
Meyer Burger Technology AG, An d. Baumschule 6-8, 09337 Hohenstein-Ernstthal, Germany

ORCiD
R. Pesch (0009-0006-5453-4325), J. Petry (0009-0004-6653-9735), J. Petermann (0009-0006-3801-2404), R. Pappenberger (0009-0001-7842-7560), T. Kuechle (0000-0002-5726-2808), X. Liu (0009-0006-3539-8591), L. Fang (0009-0003-7618-8046), S. Rafizadeh (0000-0002-9789-5917), B. Abdollahi (0000-0001-9426-1217), J. Sutter (0000-0001-5634-3449), U. Lemmer (0000-0001-9892-329X), U. W. Paetzold (0000-0002-1557-8361)

Table of Contents
Figure S1: Rendering of the tandem solar cell architecture
Figure S2: Picoliter droplet absorption for hybrid two-step inkjet printing
Figure S3. Influence of ink molarity on droplet stability
Figure S4: Comparison between LiF and $PDAI_2$:BAI surface passivation
Figure S5: Simultaneously scalable fabricated perovskite solar cell array
Figure S6: Cross-section SEM of the perovskite on textured and planar substrates
Figure S7: EQE of PSC fabricated using CsCl vs. CsBr in the first step
Figure S8: Inorganic CsCl:PbI_2 thin film deposited on textured silicon solar cell
Figure S9: Electrical performance of perovskite/silicon tandem solar cells
Figure S10: 500h MPP and JV-curves of the champion tandem solar cell
Figure S11: Laser edge isolation and single-droplet analysis
Figure S12: Inkjet-printing precision

Figure S1: Rendering of the tandem solar cell architecture

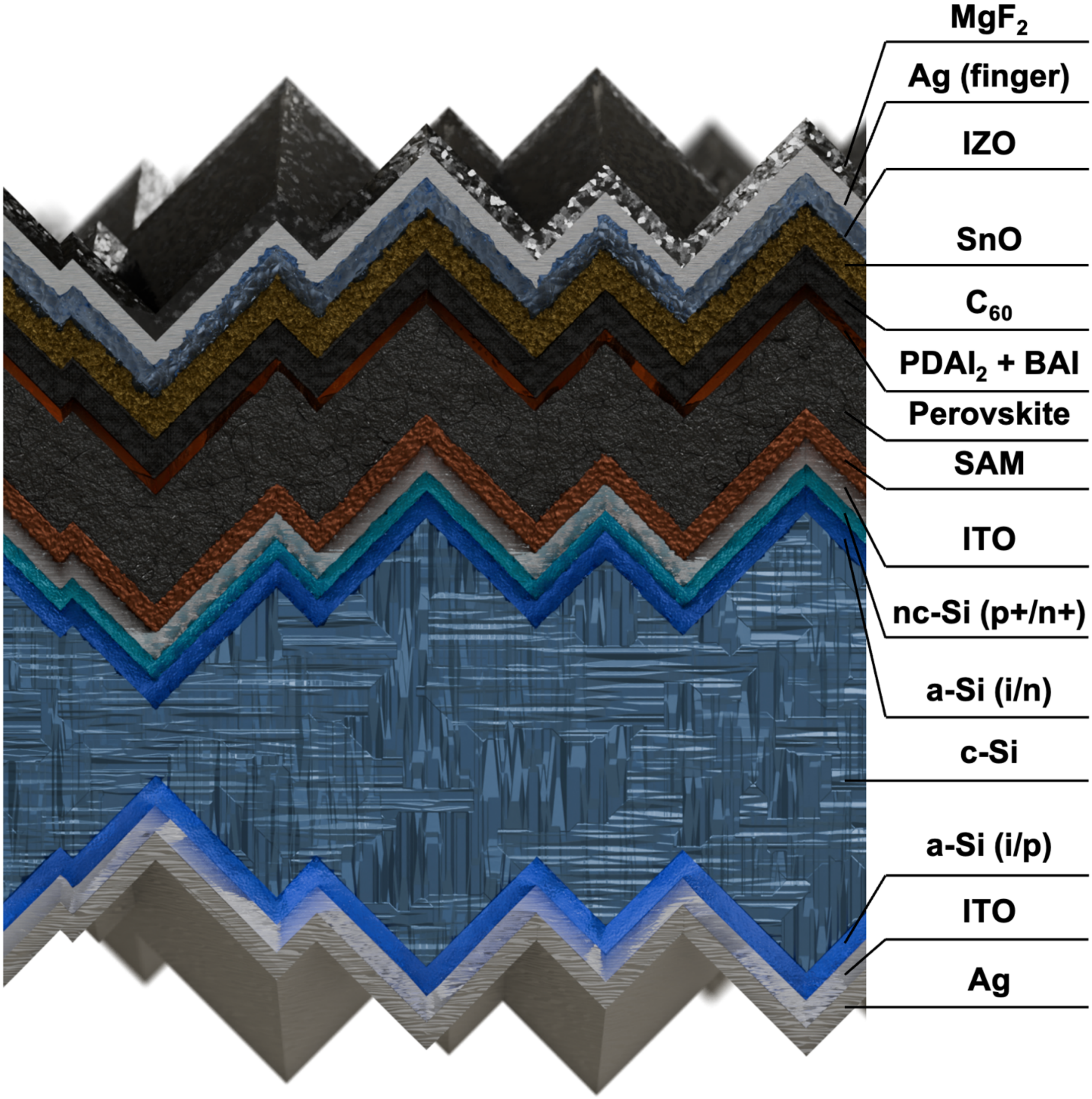

Figure S1: Rendered cross-section of the complete perovskite/silicon tandem solar cell architecture used in this work.

Figure S2: Picoliter droplet absorption for hybrid two-step inkjet printing

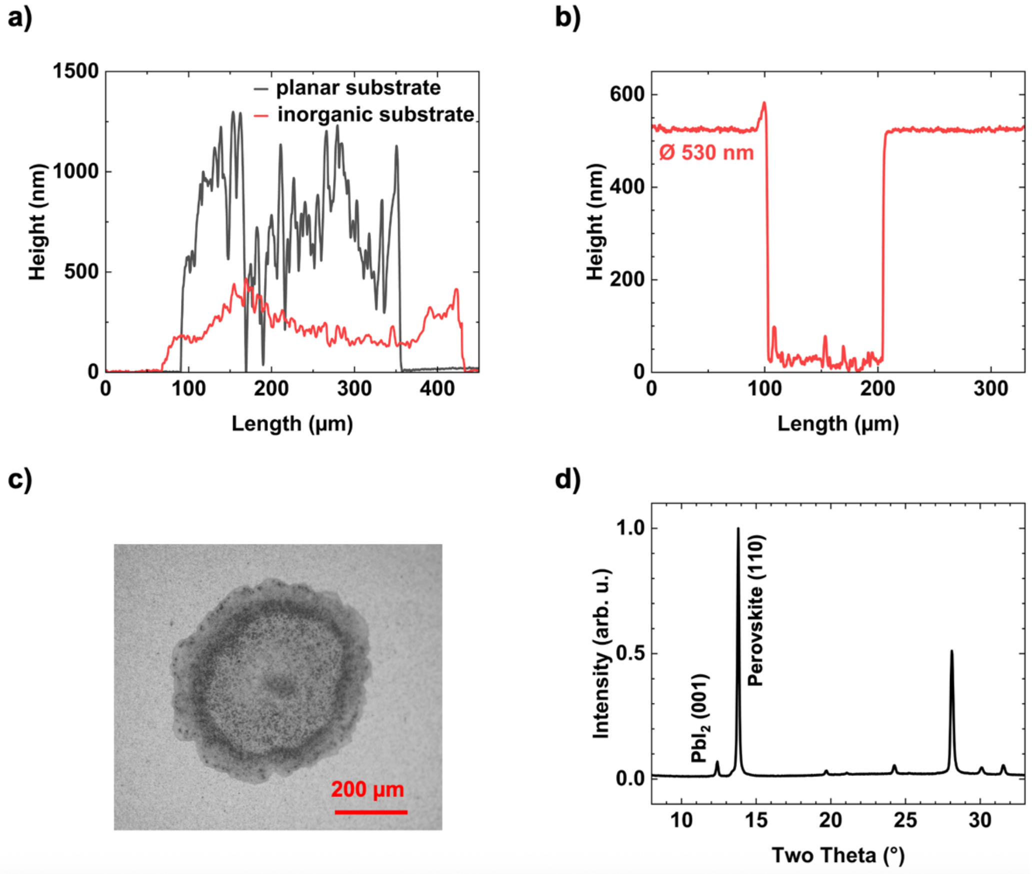

Figure S2: (a) Profilometry measurements of picoliter-size droplets after drying on a planar substrate and an evaporated inorganic thin film. (b) Profilometry measurements across a scratch of the evaporated inorganic thin films. (c) Microscope images of the picoliter-sized droplet after drying on evaporated inorganic thin films. (d) X-ray diffraction measurement of the annealed perovskite thin film.

Figure S3: Influence of ink molarity on droplet stability

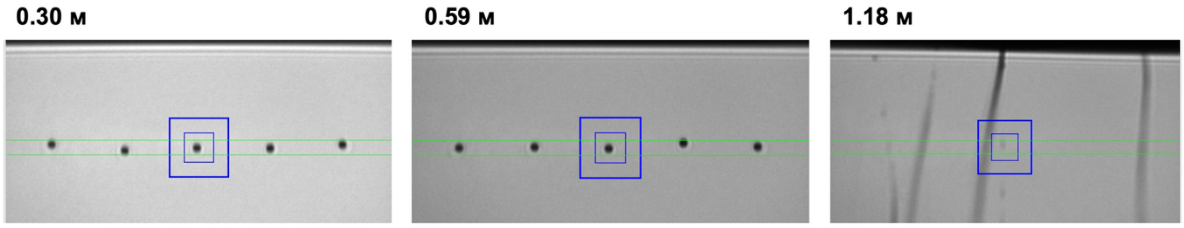

Figure S3: In situ microscopic analysis of inkjet-printed droplets at ink molarities of 0.3 M, 0.59 M, and 1.18 M.

Figure S4: Comparison between LiF and $PDAI_2$:BAI surface passivation

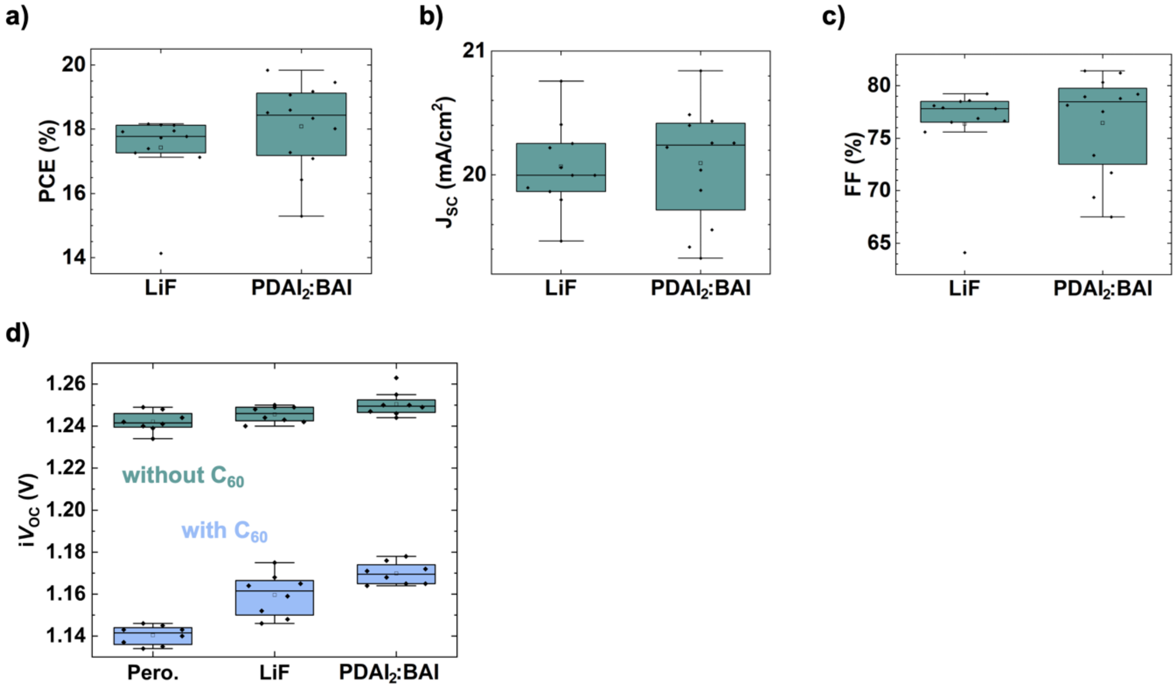

Figure S4: Distribution of power conversion efficiency (a), short-circuit current density (b), and fill factor (c) values of perovskite solar cells fabricated using lithium fluoride and propane-1,3-diammonium iodide:n-butylammonium iodide as surface passivation. d) Distribution of implied open-circuit voltage values of perovskite solar cell half-stacks using lithium fluoride and propane-1,3-diammonium iodide:n-butylammonium iodide as well as C_{60} and no C_{60} atop the perovskite

Figure S5: Simultaneously scalable fabricated perovskite solar cell array

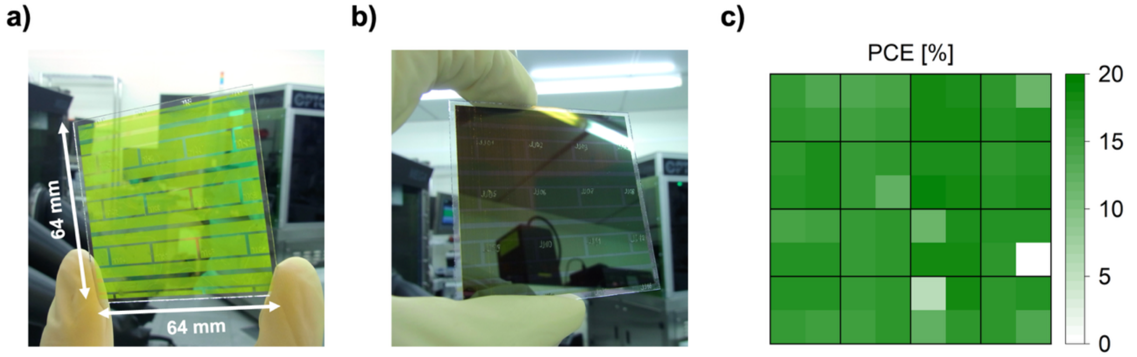

Figure S5: Images of large-area (40.96 cm²) deposited (a) inorganic and (b) perovskite thin films. (c) Distribution of power conversion efficiencies measured from spatially uniformly distributed, small-area (10.5 mm²) perovskite solar cells patterned across the substrate.

Figure S6: Cross-section SEM of the perovskite on textured and planar substrates

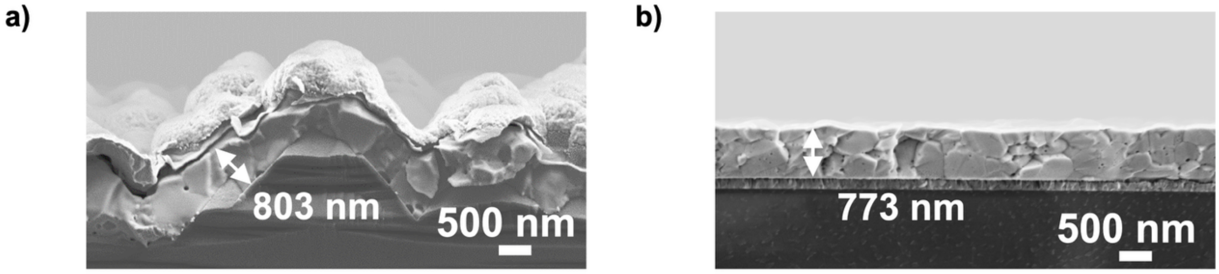

Figure S6: Cross-section scanning electron microscopy image of the perovskite on (a) textured silicon bottom solar cells using 50 nm cesium chloride + 500 nm lead iodide and (b) planar glass-indium tin oxide substrates using 24 nm cesium chloride + 240 nm lead iodide.

Figure S7: EQE of PSC fabricated using CsCl vs. CsBr in the first step

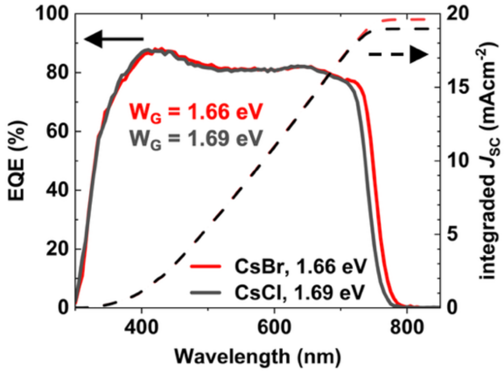

Figure S7: External quantum efficiency of single-junction perovskite solar cells fabricated by depositing 24 nm cesium bromide +240 nm lead iodide vs. 24 nm cesium chloride + 240 nm lead iodide in the first (evaporation) step.

Figure S8: Inorganic CsCl:PbI_2 thin film deposited on textured silicon solar cell

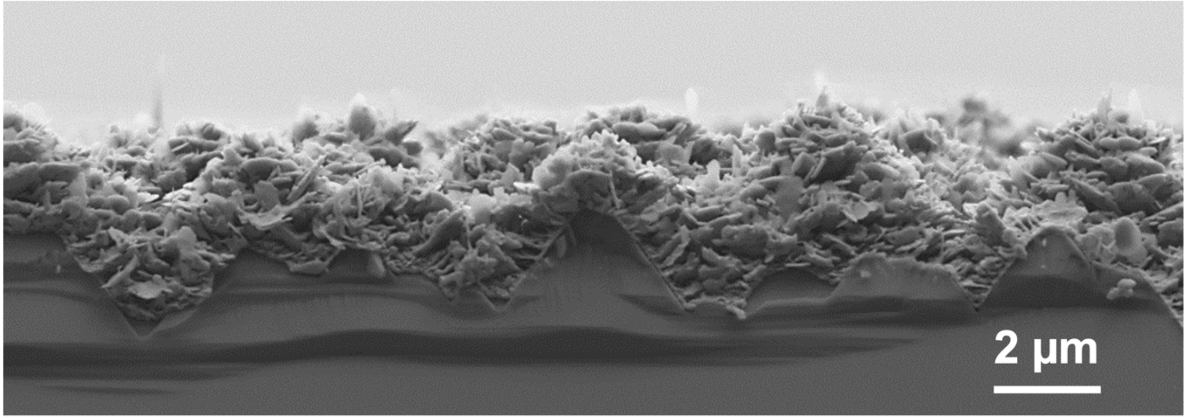

Figure S8: Cross-sectional scanning electron microscopy image of a fully textured silicon solar cell covered with the evaporated CsCl:PbI_2 inorganic thin film.

Figure S9: Electrical performance of perovskite/silicon tandem solar cells

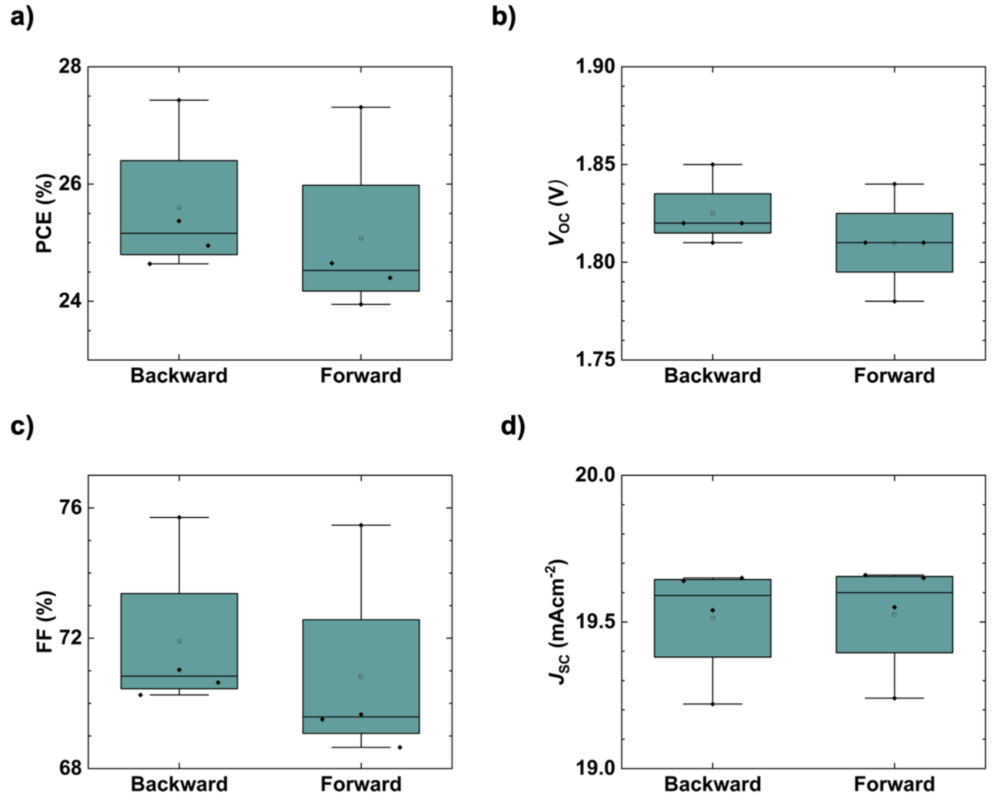

Figure S9: (a) Power conversion efficiency, (b) open-circuit voltage, (c) fill factor, and (d) short-circuit current density of the fabricated perovskite/silicon tandem solar cells.

Figure S10: 500h MPP and JV-curves of the champion tandem solar cell

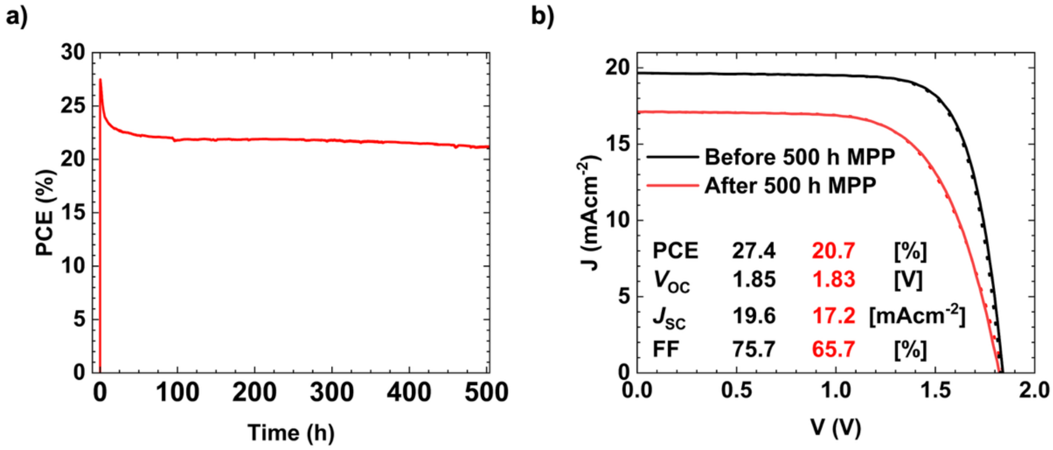

Figure S10: (a) Maximum power point tracking of the champion tandem solar cell over the course of 500 hours. b) Current density-voltage curves of the champion tandem solar cell before and after 500 hours of maximum power point tracking.

Figure S11: Laser edge isolation and single-droplet analysis

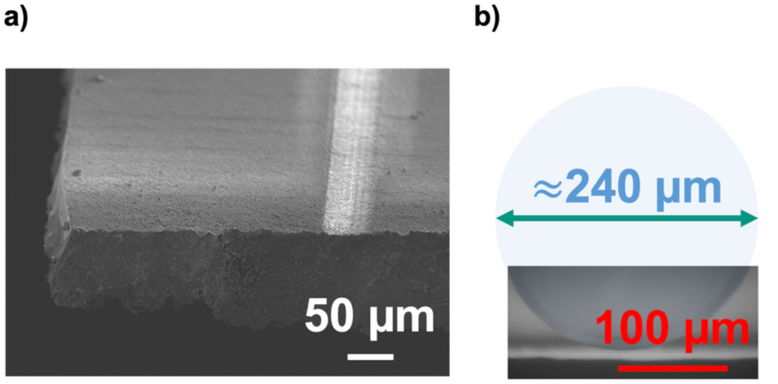

$$V_{\text{Hemisphere}} = \frac{2}{3}\pi r^3$$

$$r = \sqrt[3]{\frac{3}{2}\frac{V_{\text{Hemisphere}}}{\pi}} = \sqrt[3]{\frac{3}{2}\frac{10\ \text{pL}}{\pi}}$$

$$r = 168.4\ \mu m$$

Figure S11: (a) Exemplary replicated laser edge isolation in size and dimension of those presented in literature [21] (b) Microscope image of a (half-)droplet at the edge of a perovskite solar cell, with its digital extension used to determine its diameter. (c) Calculation of the radius of a hemisphere with a volume of 10 pL.

Figure S12: Inkjet-printing precision

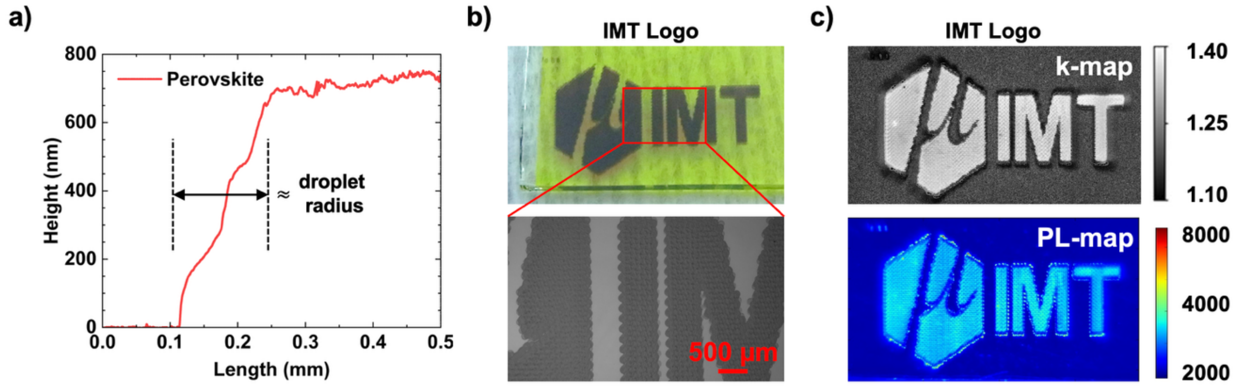

Figure S12: (a) Profilometry measurement across the edge of the annealed perovskite thin film, demonstrating spatially precise perovskite deposition up to droplet-radius distance from the substrate edge. (b) Image and microscope image of the “Institute of Microstructure” (IMT) logo printed using the hybrid two-step deposition method, highlighting precise spatial perovskite fabrication. (c) k-map and photoluminescence map of the printed IMT logo.
